# The Law as to the Commitment of the Insane, in Illinois

**Published:** 1879-02

**Authors:** 


### ARTICLE XVI.

### THE LAW AS TO THE COMMITMENT OF THE INSANE, IN ILLINOIS.

One of the first things done at the present session of the legislature of this State, should be a thorough reconstruction of the law relating to the commitment of the insane.

As the case stands at present, it is, or should be, well-known, that it is necessary to drag the insane person, male or female, young or old, or physically infirm, and their friends with them, from their homes, often at the cost of distressing inconvenience, to a public examination in a court room, amid a crowd of lawyers, on and before a jury, composed, at best, of average non-professional citizens, selected at random, largely ignorant of the subject upon which they are to pronounce. The only relief provided lies in the fact that *one physician* is required to be placed on the jury.

Besides the inconveniences of the present system there is to be objected against it, that it frequently happens that plain cases of insidious or even dangerous insanity are declared under it, to be sane, and are set free, to the annoyance and danger of friends and neighbors, or to the peril of their own lives, where the tendency is suicidal, because the jury very naturally happens to be ignorant of the more uncomman forms of mental unsoundness, or of the symptoms of such disease in its incipient stages. Then again there are but few families or persons not more or less reluctant to making a public spectacle of their friends while in a state of insanity. The present system has fostered and will continue to foster, the feeling which pervades the minds of the people, that insanity is not simply a disease,—an affliction, but a disgrace. This sentiment, quite natural under the circumstances, has often prevented, and will continue to prevent such cases from being promptly reported, and hence directly tends to prevent them from being early placed under suitable treatment. But these and other disadvantages could be quietly borne, if the present system were the best that could be devised in the interest of public and private justice. But it is so plain that there is a better way, that there is no valid excuse for delaying action.

The system we need in Illinois is the one adopted long since in many of the Eastern States and in the most enlightened foreign countries. It is usually embodied in a simple law, creating commissioners in lunacy. These are generally composed of two or more medical men of standing and experience, and a magistrate or lawyer, the appointment of the commissioners under the law being delegated to the judges of the courts within whose jurisdiction they are to act.

These commissioners may be either temporary or permanent, and be ready to act when and where the exigencies of the insane require.

The action of this commission, as suggested by Dr. Patterson, of Batavia, in his recent letter, might be supplemented by a jury trial before the courts as at present, if requested by the parties interested. The advantages of such a mode of procedure, as compared with the one at present in vogue are so manifest that it seems surprising all do not see them at once.

A more intelligent, and it may be maintained, a more just judgment would be reached than by the present system, and the Commission could act any day, instead of one day in each week, or every two weeks, and could, and would, when necessary, as it often is, visit the residence of the patient. By all means let the present law be at once modified, in conformity to more advanced ideas, and thus the present piece of clumsy legislation be forever removed from our statute books.

